# Fexinidazole and Corallopyronin A target *Wolbachia*-infected sheath cells present in filarial nematodes

**DOI:** 10.1371/journal.ppat.1012929

**Published:** 2025-09-08

**Authors:** Laura Chappell, Ricardo Peguero, William R. Conner, Sommer Fowler, Brandon S. Cooper, Kenneth Pfarr, Achim Hoerauf, Sara Lustigman, Judy Sakanari, William Sullivan

**Affiliations:** 1 Department of Molecular, Cellular and Developmental Biology, University of California, Santa Cruz, California, United States of America; 2 Department of Molecular Parasitology, New York Blood Center, Lindsley F. Kimball Research Institute, New York City, New York, United States of America; 3 Division of Biological Sciences, University of Montana, Missoula, Montana, United States of America; 4 Department of Immunology and Parasitology, Institute for Medical Microbiology, University of Bonn, Bonn, Germany; 5 German Center for Infection Research (DZIF), Partner Site Bonn-Cologne, Bonn, Germany; 6 Department of Pharmaceutical Chemistry, University of California, San Francisco, California, United States of America; University of Wisconsin-Madison, UNITED STATES OF AMERICA

## Abstract

The discovery of the endosymbiotic bacteria *Wolbachia* as an obligate symbiont of. filarial nematodes has led to antibiotic-based treatments for filarial diseases. While lab. and clinical studies have yielded promising results, recent animal studies revealed that *Wolbachia* levels rebound following treatment with the antibiotic rifampicin. Previous work revealed that a potential source of the bacterial rebound in female worms were dense clusters of *Wolbachia* in ovarian tissue. The number, size, and density of these *Wolbachia* clusters were not diminished despite antibiotic treatment. Here we define the cellular characteristics of the *Wolbachia* clusters in *Brugia pahangi* (wBp) and identify drugs that target them. We show that the *Wolbachia* clusters originate from newly formed sheath cells adjacent to the distal tip cell. The dramatically enlarged volume of a *Wolbachia*-infected sheath cell is strikingly similar to endosymbiont-induced bacteriocytes found in many insect species. Ultrastructural analysis reveals that the clustered *Wolbachia* present within the sheath cells have a distinct morphology from those present within the oocytes, and that the sheath cell membrane appears to have interdigitations with the adjacent oocyte membrane. This includes membrane-based channels that provide a connection between *Wolbachia*-infected sheath cells and oocytes. We determined that the *Wolbachia* within the sheath cells are either quiescent or replicating at a very low rate. Screens of 11 known antibiotics and other drugs revealed that Fexinidazole, Corallopyronin A and Rapamycin reduced the number of *Wolbachia* clusters infecting sheath cells but only Fexinidazole and Corallopyronin A showed a highly significant difference (p < 0.0001) compared to the control group.

## Author summary

The discovery of the endosymbiotic bacteria *Wolbachia* as an obligate symbiont of

filarial nematodes has led to antibiotic-based treatments for filarial diseases. While lab

and clinical studies have yielded promising results, recent animal studies revealed that *Wolbachia* levels rebound following treatment with the antibiotic rifampicin. Here we examine unusual dense clusters of Wolbachia present in the ovaries of filarial nematodes as they may be a potential source of the rebound. In this manuscript, we demonstrate the clusters reside in and dramatically increase the volume of a specific host cell-type, the sheath cell. We also performed a screen and identified compounds that target the clustered Wolbachia.

## Introduction

The filarial nematodes *Onchocerca volvulus, Wuchereria bancrofti, Brugia malayi* and *Brugia timori* are human parasites that cause onchocerciasis (African river blindness) and lymphatic filariasis (elephantiasis) [1]. Together these neglected diseases afflict tens of millions with many more at risk [2,3]. While drugs exist that effectively target the microfilariae and transmission, identifying effective drugs that kill adult worms has been problematic. This is of particular concern because adult *O. volvulus* live and remain fertile for 10–15 years while adult *W. bancrofti*, *B. malayi*, and *B. timori* live and remain fertile for 6–8 years. Thus, curing afflicted individuals requires a multiple-year drug regimen. In the late 1970s, cytological studies revealed the presence of a bacterium in filarial nematode hypodermal chords and germline tissues [4–6]. Subsequent sequence analysis demonstrated that the bacteria are *Wolbachia*, an endosymbiont widely distributed among a majority of insect species and most filarial nematode species [7,8]. In insects, *Wolbachia* is a facultative endosymbiont, but in filarial nematodes, *Wolbachia* is an obligate endosymbiont maintaining a significantly smaller genome.

Loss of *Wolbachia* through antibiotic treatment results in a significant reduction in fecundity and eventual death of the adult filarial worms [9,10]. This discovery led to the use of antibiotics as an effective macrofilaricidal treatment for onchocerciasis and lymphatic filariasis [11,12]. Clinical trials reveal that a 4–6 week course of antibiotics is effective at *Wolbachia* elimination and eventual killing of the adult worms after 2 years [13–15].

A recent study demonstrated that a one-week treatment of the filarial nematode *B. pahangi* with rifampicin *in vivo* resulted in a 95% reduction in *Wolbachia* (wBp) load [16]. Eight months later however, *Wolbachia* titers returned to normal levels [16]. This study also revealed the presence of dense clusters of *Wolbachia* within the ovarian tissues that were not affected despite this antibiotic treatment. Significantly, the number, size, and bacterial density of these clusters remained unchanged in the antibiotic-treated worms. Given that exposure to antibiotics had little effect on the *Wolbachia* clusters, it was postulated that these *Wolbachia* existed in privileged sites within the ovaries and that they were a possible source of the *Wolbachia* rebound [16]. As antibiotic therapy is used to treat filarial diseases and becomes more widely applied, addressing concerns regarding recrudescence and possible suboptimal treatment outcomes is critical.

**Fig 1:**
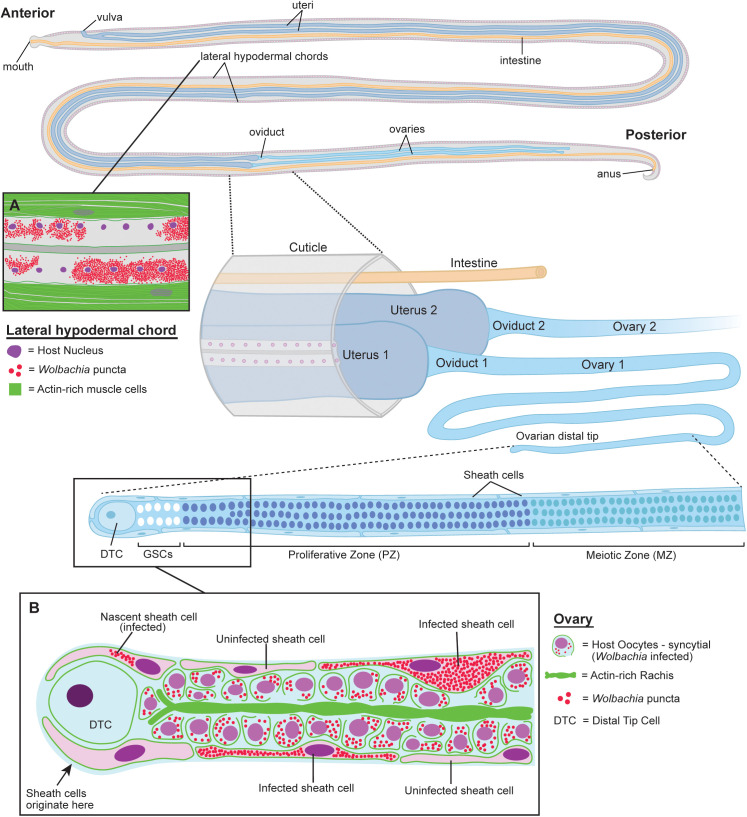
Schematic of *Wolbachia*-infected sheath cells in female *Brugia* reproductive structures. Two lateral hypodermal chords run parallel along the entire length of the nematode. These chords are syncytial with many nuclei sharing a common cytoplasm. *Wolbachia* are found in high densities in the hypodermal chords **(A)**, as well as the germline of females **(B)**. Panel A shows a zoomed depiction of a lateral hypodermal chord. Densely packed clusters of *Wolbachia* (red) are found surrounding the host nuclei (purple) within the syncytium. Panel B shows a zoomed depiction of the distal tip of one ovary. *Wolbachia* (red) reside within the syncytial oocytes, and cluster in larger masses within the ovarian sheath cells. *Brugia* adult females maintain two germlines that continuously produce thousands of eggs. This process begins at the distal tip of the ovary in which a population of germline stem cells (GSCs) associate with the Distal Tip Cell (DTC), which serves as the stem cell niche. The GSCs produce a syncytium of mitotically active oocyte nuclei that are interconnected via a central actin-rich structure, known as the rachis. As these nuclei are propelled proximally, they leave the proliferative zone and its associated rachis, cease mitotic division, form individual cells, and begin meiotic differentiation (meiotic zone). The ovary is encompassed by a flat, elongated set of cells known as the sheath cells. These cells also originate near the DTC and migrate proximally to encompass the entire ovary. In *C. elegans*, sheath cells maintain structural integrity of the oocyte and promote germline proliferation. In addition, these cells are contractile and may be involved in driving proximal migration of the cells. In this study, *Wolbachia* were found in distinct, very densely packed clusters in the sheath cells of female *Brugia pahangi* and *Brugia malayi*. Original illustration by Laura Chappell (Adobe Illustrator 28.7.1).

In the present study, we show that these *Wolbachia* clusters are located within specialized ovarian cells known as sheath cells, which we herein call *Wolbachia-*infected sheath cells. Sheath cells originate at the oocyte distal tip to ultimately encompass the syncytium of developing oocytes (Fig 1). Ultrastructural analyses using Transmission Electron Microscopy (TEM) revealed that these infected sheath cells possibly interact with adjacent oocytes via interdigitations, and that the oocytes appear connected to the ovarian rachis by cytoskeletal-like projections. Moreover, *Wolbachia* within the sheath cells appear to be morphologically distinct from those found within the oocytes and rachis. *Wolbachia*-infected sheath cells are strikingly similar to insect bacteriocytes, which are modified host cells that provide a rich, safe environment for the occupying endosymbiont [17]. Ultrastructural analyses of these sheath cells suggest that regions of these cells are connected to oocytes via the rachis and cytoskeletal-like structures, which may provide a way in which *Wolbachia* are able to traverse through the ovarian tissues

To identify new drugs that target *Wolbachia* that form large clusters within the ovarian sheath cells, we tested the efficacy of a set of repurposed drugs. These studies yielded two compounds, Fexinidazole and Corallopyronin A, that are highly potent in reducing significant numbers of *Wolbachia* within the ovarian sheath cells.

## Results

### *Wolbachia* form dense clusters within ovarian sheath cells

**Fig 2:**
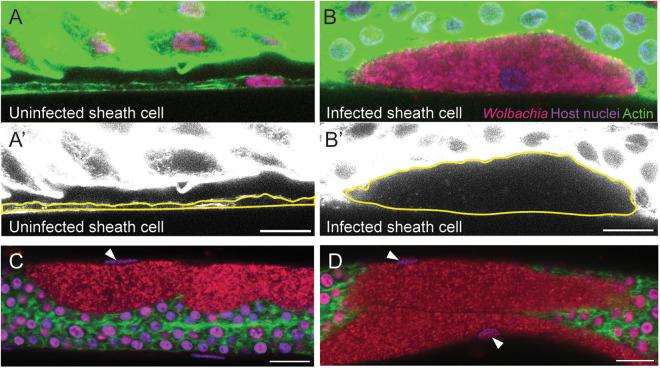
*Wolbachia* form dense clusters within ovarian sheath cells. (A-A’) Confocal microscopy of a *B. pahangi* ovary shows a sheath cell devoid of *Wolbachia*. (B-B’) A cluster of *Wolbachia* is seen inside a sheath cell. Yellow lines in lower panels (A’ and B’) outline the cortical actin of the sheath cells. **(C-D)** Infected sheath cells are greatly expanded by *Wolbachia* clusters and are associated with a single, oblong nucleus (white arrowheads). All ovarian tissues are stained with Propidium Iodide (red; *Wolbachia*), DAPI (purple; host nuclei), and Phalloidin 488 (green; actin). All scale bars are 10 µm.

Sheath cells are characterized by a single flattened nucleus (Fig 2). To image both host cells and *Wolbachia*, ovaries from *Brugia pahangi* female worms aged 126–630 days postinfection (dpi) were removed and stained with 4’,6-diamidino-2-phenylindole (DAPI), Propidium Iodide (PI), and fluorescently labeled Phalloidin [18] (Fig 2). The PI targets nucleic acids and a brief exposure preferentially stains *Wolbachia*. Unless otherwise noted, host nuclei are depicted in purple; *Wolbachia* puncta are red; and actin is green. In these infected sheath cells, the entire volume of the cytoplasm is occupied by *Wolbachia*, and the width of the sheath cells is greatly expanded compared to uninfected cells (compare Fig 2A and 2A’ to 2B and 2B’). Each cluster is localized within a host cell positioned at the outer edge of the ovary. These cells are characterized by a single flattened oblong nucleus (Fig 2C and 2D white arrows) and some clusters are juxtaposed (Fig 2D). In *C. elegans*, a single layer of sheath cells encompasses the germline during larval development and persists in the adult worm [19]. Based on the peripheral location in the gonads, the flattened shape of the nucleus and the description by Foray et al. (2018) describing similar cells in *B. malayi*, we conclude that the *Wolbachia* clusters are localized inside sheath cells.

**Fig 3:**
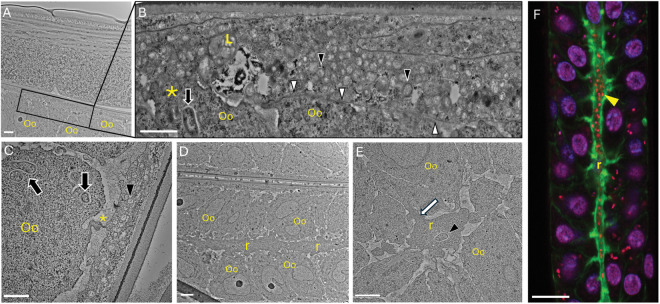
Transmission electron and confocal microscopy of adult female *B. pahangi* ovarian tissue. Adult female B. *pahangi* were cultured *in vitro* and processed for analysis by transmission electron and confocal microscopy. **(A)** Low magnification image of ovarian tissue displaying oocytes (Oo) and an infected sheath cell (boxed region). **(B)** High magnification composite image of the boxed region in panel **A.** An infected sheath cell containing a lysosome (L) and adjacent oocytes (Oo) can be seen. The sheath cell membrane (white arrowheads) is clearly visible and appears to interdigitate with an adjacent oocyte membrane (asterisk). A typical *Wolbachia* bacterium in a vacuole can be seen within an oocyte (black arrow). Numerous smaller, and electron lucent *Wolbachia* are seen within the infected sheath cell (black arrowheads). **(C)** An infected sheath cell containing numerous *Wolbachia* (black arrowhead) is seen in proximity to an oocyte (Oo) containing several *Wolbachia* (black arrows). A cellular connection extending from the oocyte to the sheath cell is visible (asterisk). **(D)** A low magnification image of the ovarian rachis (r) and surrounding oocytes (Oo). **(E)** A high magnification image of the ovarian rachis (r) surrounded by oocytes (Oo). A bacterium (black arrowhead) can be seen in the rachis in proximity to cytoskeletal projections (white arrow) that extend from the rachis into one of the adjacent oocytes. **(F)** Confocal micrograph of the ovarian rachis (r; green), surrounding oocytes with purple nuclei, and *Wolbachia* (yellow arrowhead; red puncta). Numerous *Wolbachia* can be seen throughout the rachis and within oocytes. Ovaries are stained with Propidium Iodide (red), DAPI (purple), and Phalloidin 488 (green). Scale bars: A, C-E: 2 μm; B: 1 μm; F: 10 μm.

These studies were complemented by Transmission Electron Microscopy studies of the *B. pahangi* ovarian tissue. First, we found that the flattened sheath cells are densely packed with *Wolbachia* (black arrowheads) (Fig 3A and 3B). Marked differences between *Wolbachia* within infected sheath cells and *Wolbachia* within oocytes were observed (Figs 3A, 3B, and S1). Notably, the bacteria within the sheath cells do not reside within vacuoles, are smaller and possess electron lucent inner matrices when compared to bacteria within oocytes (S1 Fig) [5]. Furthermore, we observed that the infected sheath cell membranes interdigitate in some regions with the adjacent oocytes (Figs 3B and S2), and in some instances even form connecting structures (Figs 3C and S2). These ultrastructural analyses also revealed intimate associations between the oocytes and the ovarian rachis (Figs 3D, 3E and S3), and cytoskeletal-like structures extending from the rachis into the surrounding oocytes (Figs 3E and S3). We also observed the presence of *Wolbachia* within the rachis in proximity of these structures. Confocal microscopy analyses also demonstrated the presence of numerous *Wolbachia* (red dots) within the rachis (actin in green) (Fig 3F), and similar to our ultrastructural findings, projections from the rachis that extend into the adjacent oocytes. Altogether, it is tempting to speculate that *Wolbachia* may potentially traverse through infected sheath cells, oocytes and the ovarian rachis through a series of cellular and cytoskeletal-like structural connections.

### *Wolbachia* clusters are present in nascent sheath cells located near the Distal Tip Cell

**Fig 4:**
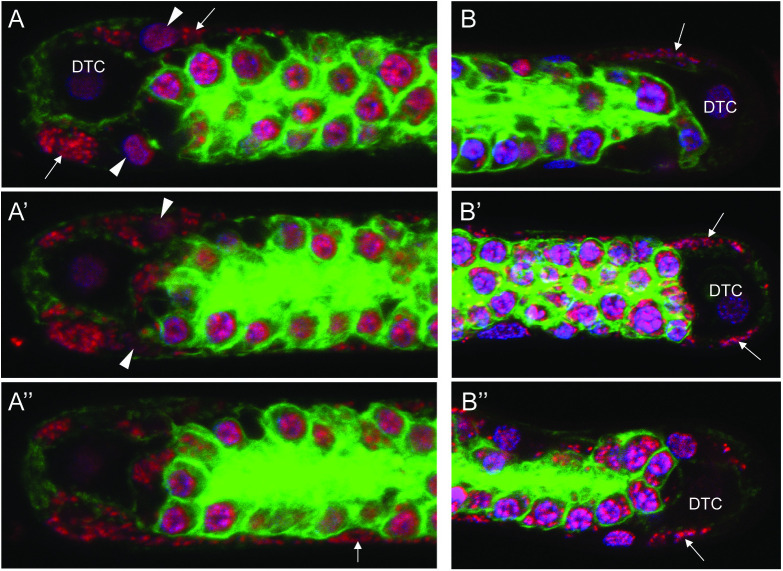
*Wolbachia* clusters are present in nascent sheath cells located near the Distal Tip Cell. Small *Wolbachia* clusters are found near, and often surrounding, the distal tip cell (DTC) of the *Brugia pahangi* ovary. (A-A”) Images represent sequential Z-stacks of a nematode ovary distal tip. Two small *Wolbachia* clusters are seen near the DTC (A; arrows). White arrowheads point to sheath cell nuclei. Imaging deeper into the tissue reveals the bacterial cluster at the bottom edge of the ovary to be part of a larger infected sheath cell (A”; arrow). Z-stack step size between images is 2.64 µm. (B-B”) Additional images of small *Wolbachia* clusters found near the DTC of other *B. pahangi* ovaries. White arrows point to *Wolbachia* clusters. Tissues are stained with Propidium Iodide (red), DAPI (purple), and Phalloidin 488 (green).

To determine where and in which cells *Wolbachia* clusters originate, we reasoned that cells of origin would contain smaller clusters and would not have undergone expansion. Analysis reveals that cells adjacent to the Distal Tip Cell (DTC) of the ovaries occasionally contain small *Wolbachia* clusters (Fig 4). In *C. elegans* the DTC serves as the niche for the germline stem cells [20]. Neighboring the DTC is the first of five pairs of sheath cells that divide, migrate, and elongate such that the entire *C. elegans* gonad is encompassed by sheath cells maintaining a distinct oblong flattened shape [19]. We suspect that the oblong flattened cells with small *Wolbachia* clusters near the *B. pahangi* DTC are nascent sheath cells. Fig 4A-4A” depict sequential confocal Z-planes cutting through the DTC of a *B. pahangi* ovary. In the top panel, arrows highlight two small clusters of *Wolbachia* in cells adjacent to the DTC, and the arrowheads highlight sheath cell nuclei (Fig 4A). Fig 4A’ and 4A” show that the clusters closely associate with the host nucleus and reside within the cell. Imaging deeper into the distal tip tissue, clusters of *Wolbachia* are more apparent and can be seen extending along the length of the expanding sheath cell. Fig 4 B-B” depict three other examples of cluster-containing cells closely associated with the DTC. Given their position within the germline tissue and oblong flattened shape, we conclude they are infected sheath cells.

### Infected sheath cells are also present in *Brugia malayi* ovarian tissues

To determine whether the *Wolbachia* clusters in the sheath cells are unique to *B. pahangi,* we examined ovaries from *B. malayi*, one of three species that infects humans and causes lymphatic filariasis [21]. The other two species which infect humans, *Brugia timori* and *Wuchereria bancrofti*, were not imaged as they cannot be cultured as adult worms for laboratory experimentation. Our analyses revealed that sheath cells containing densely packed *Wolbachia* are also present in *B. malayi* ovaries (S4 Fig). As with *B. pahangi*, the *Wolbachia* clusters are closely associated with oblong flattened nuclei (white arrowheads) in the sheath cells that encompass the germline. Similar to *B. pahangi*, *B. malayi* clusters are also found at the edges of the ovarian tissue.

### Hypodermal chord *Wolbachia,* but not oocyte or sheath cell *Wolbachia,* incorporate EdU

**Fig 5:**
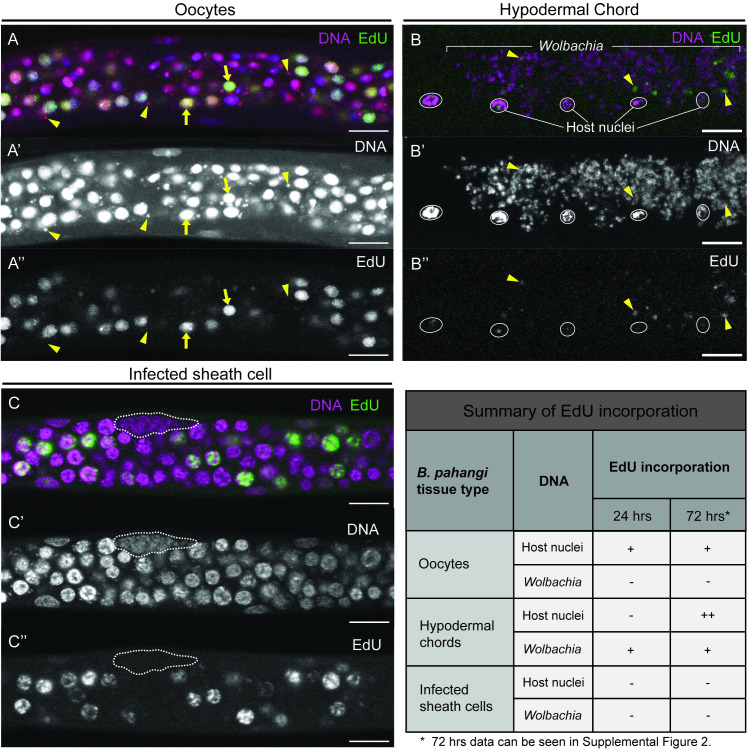
Hypodermal chord *Wolbachia,* but not oocyte or sheath cell *Wolbachia,* incorporate EdU. **(A)** Ovarian tissue of adult *Brugia pahangi* was incubated with 200 μM EdU (green) for 24 hours. DNA is co-stained with Propidium Iodide and DAPI (magenta). Mitotically active nematode host nuclei undergoing DNA replication incorporate EdU. Yellow arrows highlight two representative nuclei that have incorporated EdU. (A’) Single channel DNA imaging highlights *Wolbachia* puncta surrounding oocyte host nuclei. (A”) Single channel EdU labeling highlights host nuclei that are undergoing or have undergone DNA replication. The three yellow arrowheads highlight *Wolbachia* that have not incorporated EdU. **(B)** Hypodermal chords of adult *Brugia pahangi* were incubated with 200 μM EdU (green) for 24 hours. DNA is stained with DAPI only (magenta). Nematode host nuclei are outlined in white. All other magenta puncta are *Wolbachia*. EdU incorporation (green) can be seen amongst the *Wolbachia* puncta (arrowheads). Image represents a max projection of four Z-stacks with a step size of 0.38 μm. (B’) Single channel DNA imaging highlights the extensive distributions of *Wolbachia* in the hypodermal chords (arrowheads highlight panel B *Wolbachi*a that incorporated EdU). (B”) Single channel EdU labeling highlights host and bacterial DNA that has replicated or is undergoing replication (arrowheads highlight panel B *Wolbachia* that incorporated EdU). Given that EdU labels only a portion of the chromosome, it is likely the nuclei were undergoing replication during EdU incubation. **(C)** While EdU incorporation (green) occurred in host oocyte nuclei, EdU incorporation was not observed in *Wolbachia*-infected sheath cells after 24 hours incubation at 200 μM concentration (7 total infected sheath cells were analyzed). DNA is stained with DAPI only (magenta). White dotted line indicates the outline of the infected sheath cell. (C’) Single channel DNA imaging highlights densely packed *Wolbachia* clusters in the sheath cells. (C”) Single channel EdU labeling highlights host oocyte nuclei that have replicated or are undergoing replication. Clustered *Wolbachia* in the sheath cells remain unlabeled. For all images, EdU is visualized with Invitrogen Click-iT EdU imaging kit, Alexa Fluor 488. All scale bars are 10 μm.

To determine whether the *Wolbachia* are actively replicating in the mature infected sheath cells, we incubated ovarian tissue of adult *B. pahangi* females with 200 µM of the nucleotide analog EdU (green) for 24 and 72 hours and then fixed and stained the DNA (magenta) with a combination of PI and DAPI (Fig 5). Previous studies used this approach in *B. malayi* to examine DNA replication in the oocyte host nuclei [22]. These studies revealed that the germline stem cells adjacent to the DTC are quiescent. However, their daughter cells, destined to form oocytes, migrate anteriorly and become mitotically active in a region known as the Proliferative Zone (PZ) as evidenced by EdU incorporation [22].

Here we examined EdU incorporation in three host cell types: 1) the nuclei residing in the proliferative zone of the ovaries, 2) the hypodermal chord nuclei and 3) the sheath cell nuclei, all in parallel with EdU incorporation by *Wolbachia*. We found that approximately a third of the host nuclei in oocytes of the Proliferative Zone are EdU-positive (Fig 5A), consistent with previously published work [22] but we never observed EdU-positive *Wolbachia* in the PZ (Fig 5A, arrowheads). Small EdU-positive puncta are observed in this zone, but these all correlate with host cell nuclei rather than *Wolbachia*. In comparison, although there was little incorporation of EdU into the hypodermal chord host nuclei after a 24hr incubation (Fig 5B), there was extensive EdU incorporation after a 72hr incubation (S5A-A” Fig, yellow arrowheads). EdU-positive *Wolbachia* were also observed in the hypodermal chords (Figs 5B-B” and S5A-A”, yellow arrowheads), indicating that *Wolbachia* are replicating in the hypodermal chords, in accord with previous studies [5,23,24]. No EdU incorporation was observed in host sheath cell nuclei after 24hr and 72hr incubations, and none of the clustered *Wolbachia* were EdU positive (Figs 5C-C” and S5B-B”) suggesting that *Wolbachia* in the sheath cells and oocytes are not actively replicating or may be replicating at a significantly reduced rate compared to *Wolbachia* within the hypodermal chords.

### Identification of small molecules that target *Wolbachia* inside sheath cells

While a short-course (7-day) rifampicin treatment eliminated 95% of the *Wolbachia* in adult *Brugia*, this treatment had no effect on the number and size of the *Wolbachia* clusters [16]. In addition, rifampicin treatment did not diminish the density of *Wolbachia* within the clusters. Eight months following the 7-day course of rifampicin treatment, *Wolbachia* titers returned to normal levels. This observation led to the hypothesis that *Wolbachia* within the clusters might be a possible source of the rebound. To identify compounds that might target *Wolbachia* within these clusters, we conducted a small-scale drug screen *in vitro*.

**Fig 6:**
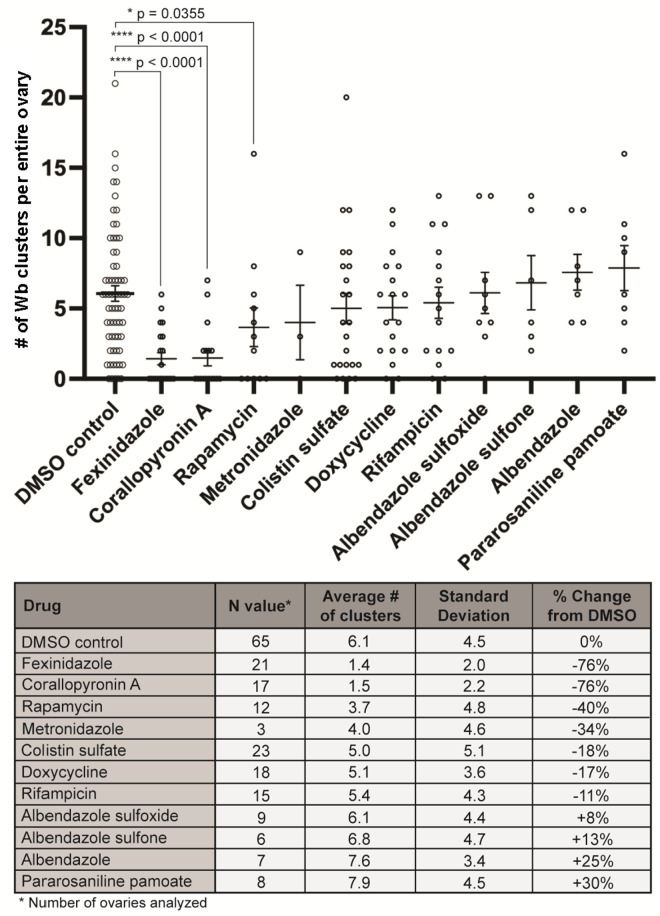
Identification of small molecules that target *Wolbachia* inside sheath cells. *Brugia pahangi* female worms were treated with small molecule drug compounds for 72 hours at a concentration of 5 µM. Ovarian tissue from drug-treated worms was dissected, fixed and stained, and the number of *Wolbachia*-infected sheath cells were scored to identify the average number found per ovary. Fexinidazole, Corallopyronin A, and Rapamycin were found to significantly reduce the number of infected sheath cells in female *Brugia* ovaries (p < 0.05). Data points represent individual ovaries analyzed. Each ovary was stained with PI and DAPI to visualize *Wolbachia*, then the entire length of the tissue was scanned from surface to surface using a confocal microscope in order to identify and score all infected sheath cells per ovary. Each compound was experimentally replicated between 2 and 5 times except for Metronidazole, which was only tested once. A Mann-Whitney statistical test was used to compare each drug compound with the DMSO control (GraphPad Prism Version 10.3.1 (509)). Main horizontal lines represent means, and error bars represent standard error of the mean, SEM.

To this end, *B. pahangi* females were incubated for three days in media containing compounds at a final concentration of 5 µM or control media (equivalent amounts of DMSO). Ovaries were dissected, fixed with paraformaldehyde, and stained with DAPI and PI. The entire volume of dissected ovaries was then scanned using confocal microscopy and the number of *Wolbachia* clusters were counted. DMSO treated worms served as a control. Because screening for drugs that eliminate the clusters using confocal microscopy is labor-intensive, we screened a limited number of compounds which consisted of a diverse set of repurposed drugs spanning a broad range of targets (S1 Table). As a positive control, we retested the effects of rifampicin, a DNA-dependent RNA polymerase inhibitor [25], on the presence of *Wolbachia* clusters. Similar to our previous results [16], while this compound effectively eliminated *Wolbachia* surrounding the ovarian sheath cells, there was no effect on the *Wolbachia* present within the sheath cells. The number of *Wolbachia* clusters in rifampicin-treated nematodes were similar to those from the DMSO-treated controls (Fig 6).

Previously we tested Albendazole and its metabolic derivatives, Albendazole sulfone and Albendazole sulfoxide [26,27]. Although these previous studies demonstrated that Albendazole and its derivatives reduced *Wolbachia* titers in insect cell lines and *ex vivo B. malayi* studies [28], these compounds did not reduce *Wolbachia* within the sheath cells. Other drugs that were not effective in this assay include Metronidazole, Colistin, Doxycycline and Pararosaniline pamoate. Rapamycin was effective in reducing abundance of *Wolbachia*-infected sheath cells by 40% (Fig 6, p = 0.0355). A summary of the compounds and their targets is provided in S1 Table. The list also includes another DNA-dependent RNA polymerase inhibitor, Corallopyronin A (CorA), and a proven potent anti-*Wolbachia* compound shown to be effective against a number of gram-negative and gram-positive bacteria [29,30]. In addition, we tested the pro-drug, fexinidazole, activated by nitroreductase [31,32].

Of all the compounds tested, Fexinidazole and Corallopyronin A were the most potent, significantly reducing *Wolbachia* within the sheath cells by 76% and 76% (p < 0.0001), respectively, compared to the DMSO control.

### Nitroreductase sequence and expression analysis

Because fexinidazole efficacy requires activation by nitroreductase, we searched for nitroreductase in publicly available *Wolbachia* genomic data*.* We found that 172 of 174 *Wolbachia* genomes—including those in the filarial nematodes *Onchocerca ochengi* (wOo_09240) [33], *Brugia malayi* (wBm_0517) [34] and *Brugia pahangi* (*w*Bp_RS00935) [35] encode a single and putatively intact nitroreductase gene copy (S6 Fig). The two exceptions are the nitroreductase copies observed in *w*Wb (wWb_CCY16_00998) associated with *Wuchereria bancrofti* [36] and *w*Ov (wOv_RS04175) associated with *Onchocerca volvulus* [37] that appear disrupted (S6 Fig). Each have a 1-bp deletion (site 358 in *w*Bm) that creates a frameshift in codon 120. This produces a premature stop codon (codon: 130, bases 387–390) in *w*Wb. *w*Ov has additional frameshifts at codons 127 and 140 due to 1-bp insertions. Translating the *w*Ov gene would produce a protein 23 residues longer than that in *w*Bm since the *w*Bm stop codon is out-of-frame. NCBI annotates the *w*Ov copy, but not the *w*Wb copy, as a pseudogene. Analysis of the two largest libraries for each of these *Wolbachia* provided no evidence of sequencing error influencing our results, with each nitroreductase gene region in these two *Wolbachia* having at least 50x coverage and with no samples having more than one read at any position in disagreement with the reference.

Analysis of existing RNA-seq data from *Brugia malayi* indicate the nitroreductase is expressed at similar levels in ovarian proliferative (PZ) and meiotic (MZ) zones and a body-wall fragment (BW) [38] (S6 Fig). The peripheral and mitotic zone dissections included the surrounding sheath cells in which the *Wolbachia*-infected sheath cells are located. Taken together, our analyses indicate most *Wolbachia* carry putatively functional copies of the nitroreductase and that this gene is expressed in multiple tissues in the *w*Bm-*Brugia malayi* system, including in peripheral and mitotic zone dissections that contained the surrounding sheath cells.

## Discussion

Studies targeting *Wolbachia*, an obligate endosymbiont bacterium in human filarial parasites, have led to promising anti-*Wolbachia* based therapies for the treatment of filarial diseases. Indeed, a number of *in vivo* animal and human clinical studies demonstrated that antibiotic treatment is an effective macrofilaricidal therapy [1,13]. However, recent studies have raised concerns that reducing *Wolbachia* titers by 95% are not always maintained after withdrawal of anti-*Wolbachia* treatment when treatment duration is short or dosing is suboptimal. For example, sub-optimal treatment with doxycycline (2 weeks rather than 4 weeks) reduced *Wolbachia* in the *Litomosoides sigmodontis* infection model, but the suppression was not sustained [39]. In this study, a short course of doxycycline was deliberately chosen to compare this regimen to that of Flubentylosin. Our previous study using the *B. pahangi* jird model of infection produced similar results [16] and despite a 95% reduction of *Wolbachia* following a 7-day rifampicin treatment, *Wolbachia* titers returned to normal pretreatment levels after 8 months.

Our previous study also revealed the presence of dense clusters of *Wolbachia* in female ovaries and that their size, number, and distribution were unaffected by exposure to rifampicin [16]. In this study we sought to further characterize these *Wolbachia* clusters and identify compounds that could target the *Wolbachia* clusters within the female *B. pahangi* ovaries *in vitro.*

In insects, some endosymbionts, including *Wolbachia*, are found within specialized cells known as bacteriocytes. These endosymbionts can induce dramatic modifications to the cells in which they reside. The bacteriocytes provide protection from the insect’s immune system and a safe cellular environment for endosymbiont replication and transmission [40,41]. In cereal weevils, endosymbionts form distinct germline and somatic bacteriocytes [42] and often increase the volume of their host cell. The bacteriocytes influence a wide array of cellular functions including metabolism, the immune response, and cell cycle, and provide a protective environment for the endosymbiont [40]. We believe that the dense clusters of *Wolbachia* present in greatly enlarged cells of female *Brugia* ovaries are similar to insect bacteriocytes. Since the clusters are found specifically in the flattened peripheral cells of the ovarian tissue, equivalent to *C. elegans* sheath cells [19], we refer to the clusters of *Wolbachia* as *Wolbachia*-infected sheath cells.

Interestingly, our ultrastructural analyses suggest that *Wolbachia* may traverse through the ovarian tissues via a series of cellular and cytoskeletal-like structural connections such as the rachis and other cellular structures. Previous studies have shown migration of *Wolbachia* from the hypodermal chords into the germline of *B. malayi* female worms and the crucial role the rachis plays in populating oocytes with *Wolbachia* and in transmitting them to the progeny [43,44]. The role of the rachis was also described wherein the endosymbionts from insect bacteriocytes were shown to escape, migrate and repopulate other tissues [40]. Studies in Drosophila demonstrated that an intact microtubule cytoskeleton and associated the associated motor proteins, kinesin and dynein were essential for maintaining Wolbachia titer and transport across the oocyte [45–47]. The potential role of cytoskeletal-like structures for such movement may provide an explanation for why Albendazole may act synergistically with some anti-*Wolbachia* drugs [48].

A striking feature of the clusters is the extensive packing of *Wolbachia* such that the volume of the sheath cell is enlarged many-fold. In addition, *Wolbachia* within the sheath cells appear to have an ultrastructurally distinct morphology compared to those within the surrounding oocytes or rachis. We also observed that the *Wolbachia-*infected sheath cells have some interdigitations with adjacent oocytes, and in some instances even form connecting structures. Our ultrastructural analyses also revealed intimate associations between the oocytes and the ovarian rachis, and the cytoskeletal-like structures extending from the rachis into the surrounding oocytes; and we observed *Wolbachia* within the rachis in proximity of these structures. Indeed, previous studies with *B. malayi* showed clear evidence of *Wolbachia* invading the germline from neighboring somatic tissues [44]. Altogether, we speculate that *Wolbachia* within the ovarian tissues may traverse through cell types via the rachis and the cytoskeletal-like structural connections or the structures that we have observed in this study. Additional ultrastructural studies will be conducted to support this hypothesis.

In *C. elegans*, sheath cells form an outer monolayer of cells that encompass the oocyte and each sheath cell contains a characteristic disc-shaped flattened nucleus [49]. *C. elegans* contains five pairs of sheath cells that provide structure to the oocyte [50] as well as regulate germline proliferation [51]. In our study, we found that *Brugia* also contain sheath cells similar to *C. elegans*, but that the filarial sheath cells surrounding the germline are infected with *Wolbachia*, making the bacteria well-positioned to reach the germline via the various cellular and structural connections.

Our EdU incorporation studies show that the *Wolbachia* within sheath cells in adult female worms (>120 days) appear to be either in a quiescent state or replicate at a very slow rate, suggesting that *Wolbachia* in the ovaries might be replenished by replicating *Wolbachia* from other sources, i.e., by *Wolbachia* residing in the hypodermal chords. A previous study revealed that *Wolbachia* first invade the somatic gonadal cells close to the ovarian distal tip cell from the hypodermis and that this movement was associated with a cortical F-actin disruption [44]. Once in the syncytial environment of the ovaries, *Wolbachia* likely rely on the rachis to multiply and disperse into the germ cells.

Clinical trials of lymphatic filariasis showed that a > 95% elimination of *Wolbachia* from female filarial worms was associated with adult worm sterility and death following 6 weeks of Doxycycline, whereas a reduction by <90% (a shorter treatment course of only 3 weeks) led to a rebound and no clear anti-parasitic activity [52,53]. Therefore, complete elimination of the symbionts is essential in eliminating transmission of the microfilarial stage of the parasite. Because *Wolbachia* were shown to rebound in *B. pahangi* following short rifampicin treatment, we were motivated to identify compounds that target *Wolbachia* in the sheath cells in an effort to prevent the reestablishment of bacteria following drug treatment. Given that the basis for the diminished response of the *Wolbachia* within sheath cells to antibiotics is unknown, we screened a diverse set of drugs with a broad range of targets. Two of the eleven drugs tested, Corallopyronin A and Fexinidazole, resulted in a highly significant reduction (p < 0.001) of *Wolbachia* within sheath cells.

Corallopyronin A is a natural product purified by liquid chromatography from extracts of the soil bacterium *Myxococcus xanthus* and targets bacterial DNA-dependent RNA polymerase in both gram-negative and gram-positive bacteria [29]. This compound has proven extremely effective at targeting *Wolbachia in vivo* [30] and is a preclinical candidate for anti-*Wolbachia* based treatment of filarial diseases. Thus, it may be preventing the transcription of *Wolbachia* genes necessary for its survival both within and outside of the infected sheath cells, and unlike other drugs, Corallopyronin A may be much more efficient at penetrating these cells. In addition, the clustered *Wolbachia* in the sheath cells likely create a hypoxic state and unlike rifampicin, Corallopyronin A is effective in a low oxygen environment [54].

Fexinidazole is an FDA-approved drug that has been particularly effective against *Trypanosoma brucei*, a blood-borne parasite and the causative agent of sleeping sickness [55]. Known as a prodrug, it is inactive until internalized by the trypanosome. Once internalized, the trypanosome enzyme nitroreductase cleaves/modifies Fexinidazole, producing toxic radicals that damage parasite DNA and protein [56]. To determine if Fexinidazole might be targeting the *Wolbachia* through a similar mechanism, we analyzed *Wolbachia* genomes for the presence of nitroreductase. We found that 172 of 174 *Wolbachia* genomes—including those in the filarial nematodes *Onchocerca ochengi* [33], *Brugia malayi* [34], and *Brugia pahangi* [35] encode a single and putatively intact nitroreductase gene copy (S6 Fig). Our analysis of existing RNA-seq data from *B. malayi* indicates the nitroreductase is expressed at similar levels proliferative and meiotic zones and a body-wall fragment [38] (S6 Fig). The proliferative and meiotic zone dissections include the surrounding sheath cells in which the *Wolbachia*-infected sheath cells are located. While it is not yet possible to determine whether the nitroreductase gene is being expressed in *Wolbachia*-infected sheath cells, we hypothesize such expression as a possible mechanism of action for the effect of Fexinidazole on this *Wolbachia* population.

To our knowledge, this is the first study that characterizes *Wolbachia*-infected sheath cells in the filarial nematode, *Brugia pahangi* and explores the effects of drugs on *Wolbachia*-infected sheath cells. This study provides information for future studies, including IC_50_s determinations which could address the possibility that the Eagle Effect (the phenomenon in which higher concentrations of drug correlate with increased titers of bacteria) might be at play, given that compounds were tested only at a single concentration [57,58]. It is possible that some of the compounds, which showed little or no effect at 5 µM might be more effective at higher doses or even at lower concentrations. In addition to extending the *in vitro* assays, *in vivo* studies are critical to further assess Corallopyronin A and Fexinidazole as potential anti-filarial therapies. Other important questions remain regarding the mechanism of rebound observed in our previous study [16]. Recent studies have explored the role of the host environment in the differential response of somatic and germline tissue to the host’s immune response and antibiotic treatment [59]. Future studies are planned to address the nature of the rebound, i.e., tracking the populations of *Wolbachia* from the hypodermal chord or other tissues over a time course following drug removal in both male and female *B. pahangi*.

## Conclusion

We have characterized *Wolbachia* clusters that are found within the ovaries of the filarial nematode *B. pahangi* and refer to these as *Wolbachia*-infected sheath cells. Ultrastructural analyses of these sheath cells suggest that regions of these cells are connected to oocytes via rachis and cytoskeletal-like structures, which may provide a way in which *Wolbachia* are able to traverse through the ovarian tissues and possibly repopulate other tissues. Our EdU studies suggest that *Wolbachia* inside sheath cells are not replicating or replicate at a very low rate which presents a challenge when attempting to identify drugs that can eliminate *Wolbachia* from the sheath cells. Eleven repurposed drugs were tested to assess their effectiveness in reducing the clusters of *Wolbachia* within sheath cells *in vitro*; two drugs, Corallopyronin A and Fexinidazole were particularly potent in reducing the *Wolbachia* clusters. This is the first study that explores the effects of drugs on *Wolbachia*-infected sheath cells and provides information for future *in vivo* studies to further examine these and other drugs as potential anti-filarial therapies.

## Methods

### Parasite material

Live *B. pahangi* and frozen *B. malayi* worms, harvested from infected jirds (*Meriones unguiculatus*), were supplied by the NIAID/NIH Filariasis Research Reagent Resource Center (FR3, Athens, GA, USA, www.filariasiscenter.org) via the Biodefense and Emerging Infections Research Resources Repository (BEI Resources, Manassas, VA, USA). The ages of the worms ranged from 126 to 630 dpi. Upon delivery, live worms were placed in an incubator at 37˚C with 5% CO_2_ to recover overnight from shipping. Drug treatments or EdU incubations were performed the following day with fresh *Brugia* media: 80% RPMI (Gibco), 10% FBS (Life Technologies) and 10%DMEM (Coring). No antibiotics nor antifungals were added.

### Drug treatments

Stock concentrations of 10 mM were created for all drug solutions using DMSO solvent (Sigma-Aldrich). Stock concentrations of the tested drugs, listed in S1 Table, were diluted to a working concentration of 5 µM in *Brugia* media. All stock concentrations were stored in the dark at -80˚C and freeze-thaw cycles did not exceed four cycles for any drug tested nor used beyond 18 months from the time of preparation. Worms were treated for 72 hours in 8 mL of drug-media or control media (equivalent amounts of DMSO) in 6-well plates at 37˚C and 5% CO_2_. Media was replaced daily until the final day when worms were collected in 1.5 mL of media and immediately frozen at -80˚C for later immunostaining.

Because the drug assays involve screening the entire volume of the ovaries for clusters using confocal microscopy and are extremely labor intensive, 11 compounds were selected and screened at a single dose and a single timepoint, rather than testing 2 or 3 compounds at multiple doses. Previous cell-based screens identified compounds with a IC_50_ < 1 µM as potent anti-wolbachial compounds [60], and we therefore estimated that a five-fold increase in this dosage (5 µM) would detect potential compounds that targeted *Wolbachia* in the clusters.

### Tissue collection

For confocal analysis of germline tissue, frozen worms were thawed at room temperature and immediately fixed in 3.2% paraformaldehyde (Electron Microscopy Sciences, 15714) in PBS for 25 minutes. Worms were rinsed twice with PBS and ovaries were dissected in PBS using microdissection tweezers. Briefly, at the posterior third of the length of each worm, the outer cuticle layer was gently broken by gripping and pulling the tweezers in opposite directions at this location. Holding the tissue anterior to the cut with one tweezer, the posterior end of the tail was pulled with the other tweezer until the cuticle was completely removed, exposing the two germlines and the intestine of the worm. Dissected germlines were collected in PBS-T (1X PBS with 0.1% Triton X-100) for subsequent immunostaining.

For electron microscopy analysis of germline tissue, live worms were immediately plunged into fixative made of 2% glutaraldehyde (VWR International, 16019) and 2.5% paraformaldehyde (Electron Microscopy Sciences, 15714) buffered with 0.1M sodium cacodylate (Electron Microscopy Sciences, 11654). Worms were fixed at room temperature for 2 hours and dissected in fixative during this incubation time. To dissect the tissue for TEM analysis, a razor blade was used to cut several 1mm posterior sections of the worm containing ovarian distal tips with attached cuticle tissue. Dissected sections, still in fixative, were placed in 4˚C and kept cold until use.

### Fluorescent staining

Dissected germline tissue was incubated with RNAse A (10 mg/mL in PBS) overnight at room temperature. Tissue was rinsed twice with PBS-T, then incubated with 1X Phalloidin 488 (Thermo Fisher) in PBS-T overnight at room temperature, protected from light. Tissue was rinsed twice with PBS-T, then stained with Propidium Iodide (Thermo Fisher) at a dilution of 1:100 in PBS-T (stock concentration of 1 mg/mL) for 25–30 seconds and immediately rinsed twice with PBS-T. Samples were mounted on glass slides with Vectashield Mounting Media with DAPI (Vector Labs) and imaged via confocal microscopy.

### EdU assay

5-ethynyl-2’-deoxyuridine (EdU) is a thymidine analog that incorporates into actively replicating DNA. We used the Click-iT^®^ EdU Imaging Kit with Alexa Fluor^®^ 488 from Invitrogen to perform our EdU assays. Adult female *B. pahangi* were incubated in 200 µM of EdU in *Brugia* media for 24 hours or 72 hours. Live worms were frozen and stored at -80˚C for subsequent processing.

Frozen worms were thawed at room temperature and immediately fixed, dissected, and stained according to Invitrogen’s protocol. Briefly, worms were fixed with 3.7% formaldehyde (Electron Microscopy Sciences) in PBS for 25 minutes. Worms were rinsed twice in PBS, and germline tissue was dissected as described above in PBS. Germline tissue was collected in 0.5 mL Eppendorf tubes and washed with 3% BSA in PBS. To permeabilize membranes, tissue was incubated in 0.5% Triton X-100 in PBS for 20 minutes at room temperature. After two rinses with 3% BSA, tissue was incubated in Click-iT^®^ reaction cocktail made fresh according to protocol for 30 minutes. After two rinses with 3% BSA, tissue was mounted onto glass slides with Vectashield Mounting Media with DAPI (Vector Labs) and imaged via confocal microscopy.

### Confocal microscopy analysis

Confocal microscopy was used to assess the effects of 11 drugs on the abundance of *Wolbachia* clusters in sheath cells from the ovaries of *B. pahangi*. The number of *Wolbachia* clusters was determined by capturing Z-stacks to measure the entire volume of each ovary. Images were obtained with an inverted laser scanning Leica SP5 confocal microscope using a 63x/1.4-0.6 NA oil objective and a resonant scanner (8000 Hz). For *Wolbachia*-infected sheath cell analysis, ovaries were scanned in search of *Wolbachia* clusters via direct observation through eyepieces using a DAPI filter. Due to the differential staining of PI and DAPI, *Wolbachia* are seen as red puncta through the microscope eyepiece, making identification of clustering bacteria clear. For every assay, control worms were processed side-by-side with the drug treated worms. The entire ovary from treated worms (n = 6–23) and DMSO treated worms (n = 65) was scanned from the top surface to the bottom surface along the entire length of the ovary in order to identify and score all infected sheath cells per ovary. Each infected sheath cell was scanned in the Z- as well as X- and Y- planes. Since only the positively infected sheath cells from both treated and DMSO control groups were analyzed, sheath cells lacking *Wolbachia* had no influence in the analyses. In total, 1,061 clusters were scored from 204 ovaries. All clusters filled the sheath cells and no obvious differences in the density of *Wolbachia* was observed for each cluster. Digital images were processed and analyzed using ImageJ 1.54d software.

### Transmission electron microscopy analysis

Six *Brugia* distal tips were received in 2% glutaraldehyde/2.5% paraformaldehyde buffered with 0.1 M sodium cacodylate. Samples were washed in buffer, and post-fixed with 1% osmium tetroxide. Samples were washed in buffer again before dehydration in an increasing ethanol series that included *en bloc* uranyl acetate staining at 70% ethanol. Following dehydration, samples were desiccated with propylene oxide and infiltrated in a 1:1 propylene oxide:epoxy resin mixture. Infiltration was continued in pure epoxy resin before embedment in pure epoxy resin at 60˚C for 48 hours. Polymerized blocks were trimmed with a razor blade and ultrathin sections were collected on formvar/carbon-coated 100 mesh grids using an RMC Boeckler Powertome and a Diatome diamond knife. Sections were contrasted with uranyl acetate and Reynolds’ lead citrate and imaged in a Tecnai G2 Spirit TEM equipped with an AMT camera and imaging software. Micrograph contrast and brightness was balanced using ImageJ software.

## Statistical analysis

Data from the drug-treatment experiments were tested for normality using the Shapiro-Wilk test and determined to significantly depart from normality. Therefore, the nonparametric equivalent of the one-way ANOVA test, the Kruskal-Wallis test, was performed on the entire dataset. It was determined that at least one sample was statistically significant from the others (p = 0.0002). A subsequent Mann-Whitney test was performed comparing the DMSO control with each drug treatment. Analyses were performed using GraphPad Prism Version 10.3.1 (509).

### Nitroreductase sequence and expression analysis

We extracted the only gene annotated as a nitroreductase from NCBI’s *w*Oo annotation (wOo_09240) [32]. We then extracted the wOo_09240 nitroreductase domain using its UniProt annotation and used tblastn to BLAST it against all *Wolbachia* sequences in NCBI available as of Oct 11, 2023. In two cases (*w*Ov and *w*Wb) the nitroreductase gene appeared disrupted. To investigate the potential for sequencing error to influence our inference, we evaluated the two largest libraries from different studies on SRA for *O. volvulus* (ERR070028, SRR1652646) and *W. bancrofti* (SRR26220905, SRR8188291). We aligned reads to the respective host and *Wolbachia* reference genomes using minimap2 v. 2.28 [61], assessed coverage and visually inspected the nitroreductase gene region for anomalies such as reads disagreeing with the reference an a position or the presence of a highly supported indel compared to the reference.

We obtained the RNA seq data produced by Chevignon et al. (2021) that measured *w*Bm expression in three *B. malayi* tissues: the PZ, the MZ, and the BW described above. Following the methods of Chevignon et al. (2021), we aligned reads to the *B. malayi* mitochondria and *w*Bm *Wolbachia* genome with bowtie2 v2.5.1 [62] We used the following options: –very-sensitive-local, —no-mixed, —no-discordant. We used featureCounts from subread v1.6.2 to count hits to each transcript [63]. Normalization and differential expression analysis were performed with the R package DESeq2 v1.42.0 [64].

## Supporting information

S1 Table List of repurposed drugs screened for anti-wolbachial activity in infected sheath cells. (PDF)

S1 Fig *Wolbachia* within sheath cells display distinct ultrastructural morphology compared to *Wolbachia* within oocytes. (A-B) Additional examples of *Wolbachia* (yellow arrows) residing within oocytes. The bacteria are within vacuoles and their inner matrices are granular and electron dense. (C-D) Additional examples of *Wolbachia* (red arrows) clusters within infected sheath cells. The bacteria within the sheath cells do not reside within a vacuole, and their inner matrices appear electron lucent. Scale bars: A-C 500 nm; D 100 nm.
(TIF)

S2 Fig Infected sheath cells and adjacent oocytes interdigitate and form connecting structures. (A) A left-side extended photo montage of an infected sheath cell (ShC) and adjacent oocytes (Oo) from Fig 3B highlighting the sheath cell membrane using yellow dashed lines and marking (yellow arrowheads) a portion of the membrane where it is discontinuous and appears to interdigitate with an adjacent oocyte. (B-C) Additional examples of sheath cells (ShC) displaying discontinuous regions of the membrane (yellow arrowheads) that interdigitate with the adjacent oocytes (Oo). Scale bars represent 1µm.
(TIF)

S3 Fig Cytoskeletal-like projections are present in the ovarian rachis in proximity of the surrounding oocytes. (A-B) Additional examples of regions of ovarian rachis (r) displaying cytoskeletal-like projections (yellow arrowheads) that extend into the surrounding oocytes (Oo). (C-C’) Consecutive serial sections, approximately 70 nm apart, of ovarian rachis (r), showing in C’ more prominently the presence of cytoskeletal-like projections (yellow arrowheads), even though the two sections are from the same region. (N), oocyte nuclei. Scale bars represent 1µm.
(TIF)

S4 Fig Infected sheath cells are also present in *Brugia malayi* ovarian tissues. (A-C) *Wolbachia* clusters are found in one of the species of filarial nematode that infects humans, *Brugia malayi*. Nematode germline tissue is stained with Propidium Iodide (red), DAPI (purple), and Phalloidin 488 (green). White arrows point to *Wolbachia* clusters in infected sheath cells. White arrowheads point to sheath cell nuclei. All scale bars are 10µm.
(TIF)

S5 Fig *Wolbachia* in the infected sheath cells do not incorporate EdU after 72 hours. (A-A”) Hypodermal chords of adult *Brugia pahangi* were incubated with 200 µM EdU for 72 hours. Nematode host nuclei are outlined in white. All other magenta puncta are *Wolbachia.* EdU incorporation can be seen amongst the *Wolbachia* puncta (yellow arrowheads). Image represents a max projection of four z-stacks with a step size of 0.38 µm. DNA is stained with DAPI only. (B-B”) Ovarian tissue of adult *Brugia pahangi* was incubated with 200 µM EdU for 72 hours. EdU does not incorporate in *Wolbachia*-infected sheath cells (white dotted outline; a total of 7 infected sheath cells were analyzed). The boxed region is enlarged in the inset to the right. Nematode host oocyte nuclei incorporate EdU, but *Wolbachia* puncta do not (yellow arrowheads point to three representative *Wolbachia* puncta). DNA is stained with DAPI only. For all images, EdU is visualized with Invitrogen Click-iT EdU imaging kit, Alexa Fluor 488. All scale bars are 10 µm.
(TIF)

S6 Fig The nitroreductase gene (ntr) is expressed by *Wolbachia* in the nematode germline. The nitroreductase gene (ntr) is expressed at similar levels by *w*Bm in three *B. malayi* host tissues: the proliferative zone (PZ), the meiotic zone (MZ), and the body wall (BW). (A) Raw counts of ntr and actin-like gene Wbm0154 output by featureCounts for the PZ dissection. While technical aspects can affect count number, this illustrates that the ntr gene is expressed, but at a lower level relative to actin-like Wbm0154. (B-D) Volcano plots showing similar expression of ntr across the three tissues. The Y axis is -log_10_ false discovery rate (FDR) and the X axis is log_2_ (fold change). The ntr gene is denoted in each plot. Sequencing library data were obtained from Chevignon et al. (2021). The solid black horizontal line and the vertical dashed lines denote the criteria used in Chevignon et al. (2021) for their assessment of differential gene expression: llog_2_(fold change)l > 2 with an FDR < 0.01. The ntr gene, like the majority in the analysis of Chevignon et al. (2021), is not differentially expressed between tissues. The relative expression of the nitroreductase is similar to expression of several other genes that include nusB (a transcription termination factor), ribosomal protein L17, and tRNA-Thr. Table indicates conserved nitroreductase genes found in the genome sequences of nine filarial nematode species.
(PDF)
