# Supplementary material for: Fexinidazole and Corallopyronin A target Wolbachia-infected sheath cells present in filarial nematodes
Source: PLoS Pathog. 2025 Sep 8;21(9):e1012929. doi: 10.1371/journal.ppat.1012929 (PMC12443271; doi:10.1371/journal.ppat.1012929)
Supplement: S1 Table — (PDF) [file ppat.1012929.s001.pdf]

Drug	Mechanism/Target	Reference	Manufacturer (Cat. No.)
**Albendazole**	Nematode beta-tubulin	Borgers, et al. 1975	Thermo Scientific (H25925-22)
**Albendazole sulfone**	Nematode beta-tubulin	Marriner, et al. 1980	Fisher Scientific (501454432)
**Albendazole sulfoxide**	Nematode beta-tubulin	Marriner, et al. 1980	Fisher Scientific (501415383)
**Colistin sulfate**	Phospholipid A, outer membrane	Velkov, et al. 2013	Fisher Scientific (50247446)
**Corallopyronin A**	DNA-dependent RNA polymerase	Krome, et al. 2022	IMMIP (N/A)
**Doxycycline**	Bacterial 30S ribosomal subunit	Nguyen, et al. 2014	Fisher BioReagents (BP26531)
**Fexinidazole**	Prodrug: nitroreductase-activated	Deeks, 2019	Med Chem Express (HY-13801)
**Metronidazole**	Prodrug: oxidoreductase-activated	Dingsdag and Hunter, 2018	WW Grainger Inc. (30TZ38)
**Pararosaniline pamoate**	Heat Shock Protein 90	Shahinas, et al. 2015	Sigma-Aldrich (SIAL-P3750)
**Rapamycin**	mTOR	Ballou and Lin, 2008	Fisher Scientific (507513705)
**Rifampicin**	DNA-dependent RNA polymerase	Mosaei, et al. 2019	TCI America (R007925G)
