# Supplementary figures and images for: Fexinidazole and Corallopyronin A target Wolbachia-infected sheath cells present in filarial nematodes
Source: PLoS Pathog. 2025 Sep 8;21(9):e1012929. doi: 10.1371/journal.ppat.1012929 (PMC12443271; doi:10.1371/journal.ppat.1012929)

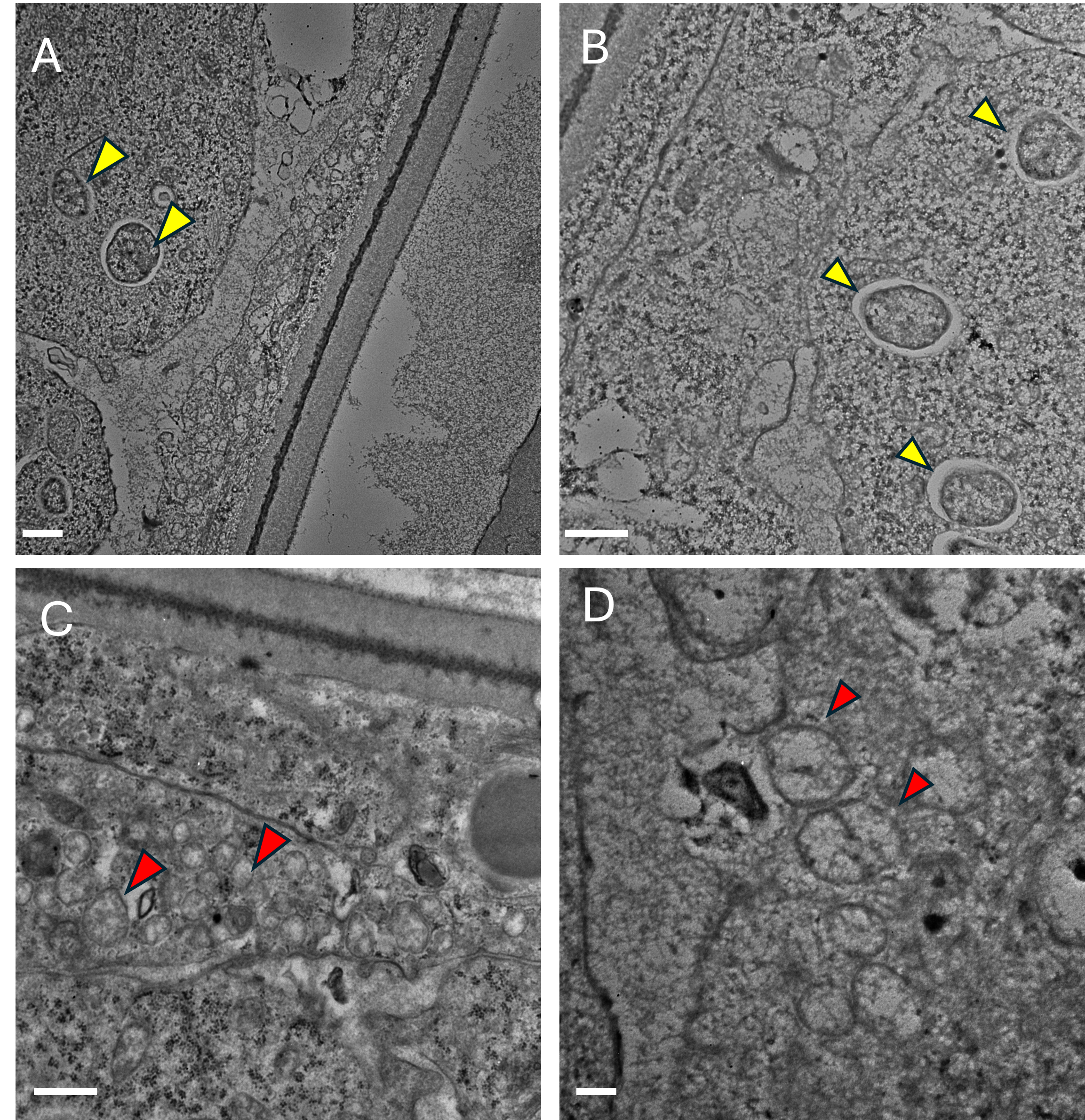

Supplement: S1 Fig — (A-B) Additional examples of Wolbachia (yellow arrows) residing within oocytes. The bacteria are within vacuoles and their inner matrices are granular and electron dense. (C-D) Additional examples of Wolbachia (red arrows) clusters within infected sheath cells. The bacteria within the sheath cells do not reside within a vacuole, and their inner matrices appear electron lucent. Scale bars: A-C 500 nm; D 100 nm. (TIF) [file ppat.1012929.s002.tif]

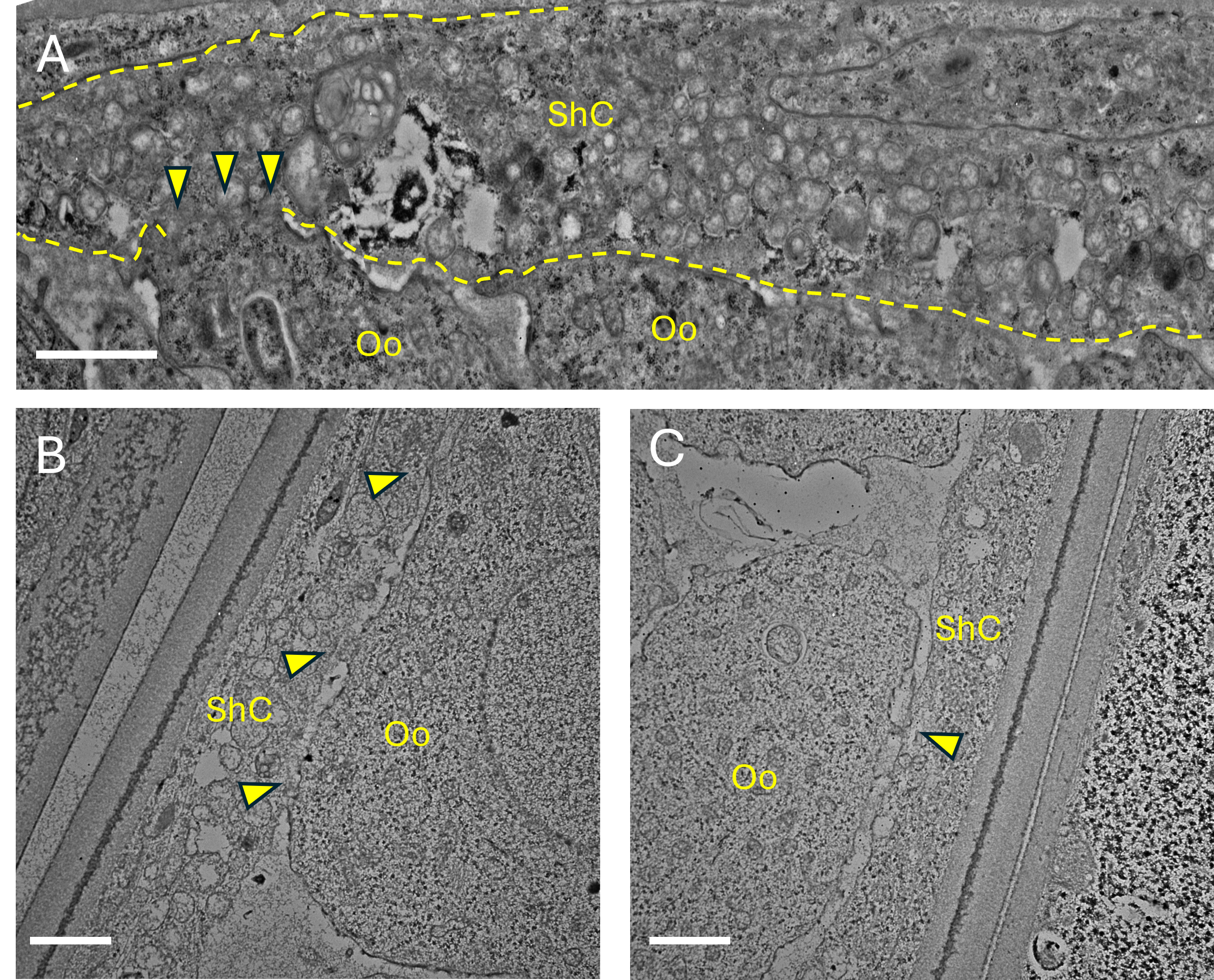

Supplement: S2 Fig — (A) A left-side extended photo montage of an infected sheath cell (ShC) and adjacent oocytes (Oo) from Fig 3B highlighting the sheath cell membrane using yellow dashed lines and marking (yellow arrowheads) a portion of the membrane where it is discontinuous and appears to interdigitate with an adjacent oocyte. (B-C) Additional examples of sheath cells (ShC) displaying discontinuous regions of the membrane (yellow arrowheads) that interdigitate with the adjacent oocytes (Oo). Scale bars represent 1µm. (TIF) [file ppat.1012929.s003.tif]

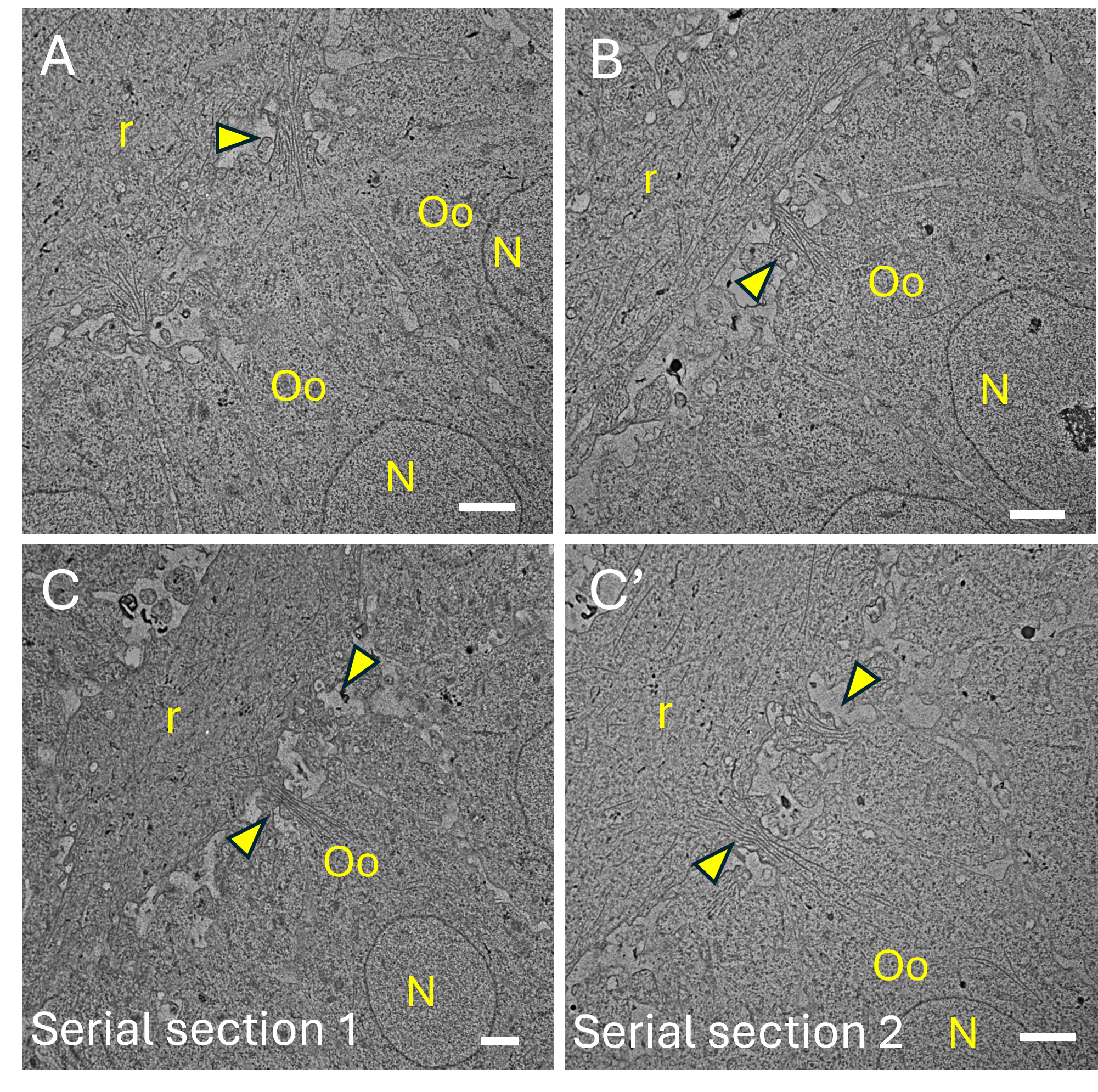

Supplement: S3 Fig — (A-B) Additional examples of regions of ovarian rachis (r) displaying cytoskeletal-like projections (yellow arrowheads) that extend into the surrounding oocytes (Oo). (C-C’) Consecutive serial sections, approximately 70 nm apart, of ovarian rachis (r), showing in C’ more prominently the presence of cytoskeletal-like projections (yellow arrowheads), even though the two sections are from the same region. (N), oocyte nuclei. Scale bars represent 1µm. (TIF) [file ppat.1012929.s004.tif]

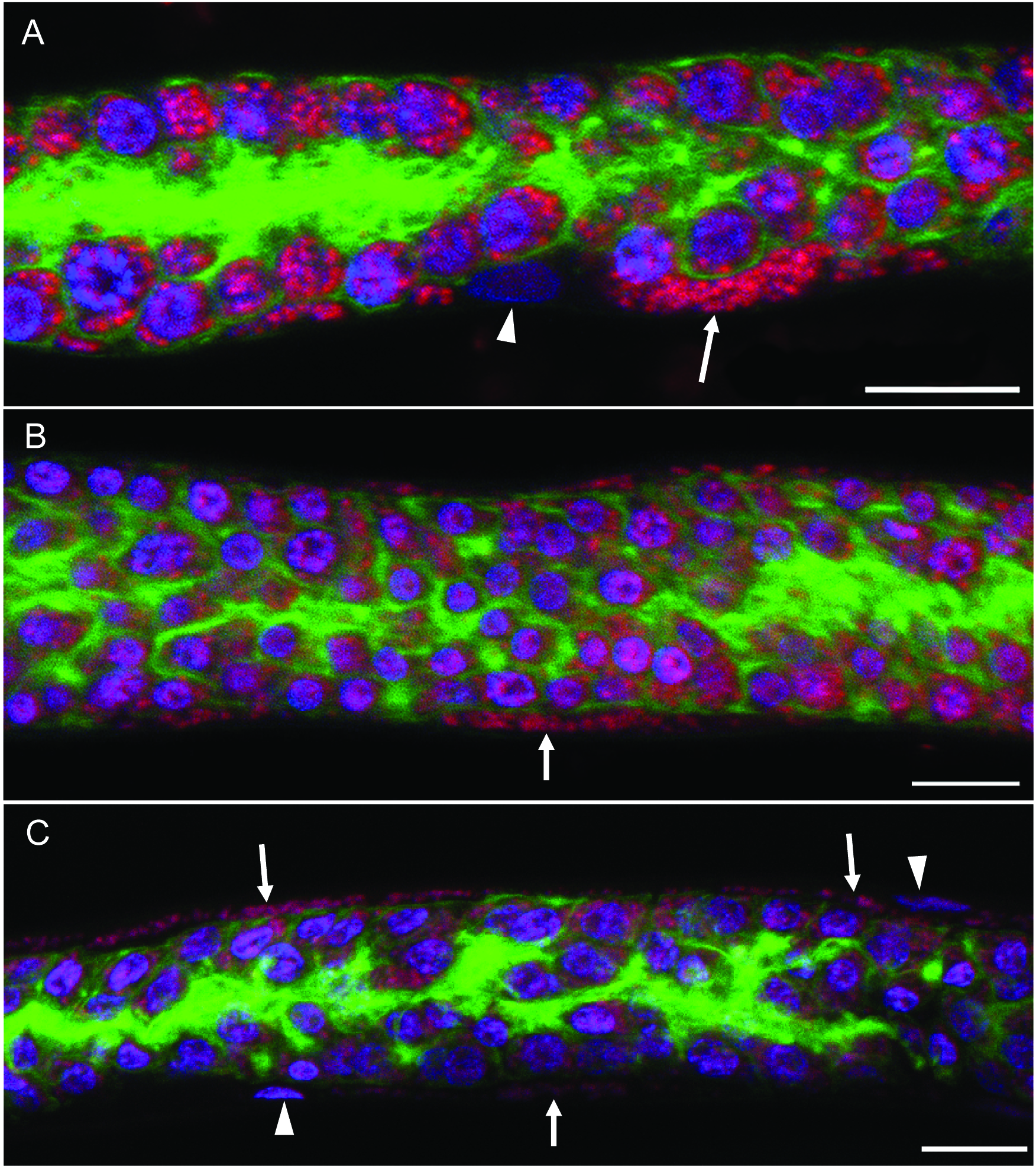

Supplement: S4 Fig — (A-C) Wolbachia clusters are found in one of the species of filarial nematode that infects humans, Brugia malayi. Nematode germline tissue is stained with Propidium Iodide (red), DAPI (purple), and Phalloidin 488 (green). White arrows point to Wolbachia clusters in infected sheath cells. White arrowheads point to sheath cell nuclei. All scale bars are 10µm. (TIF) [file ppat.1012929.s005.tif]

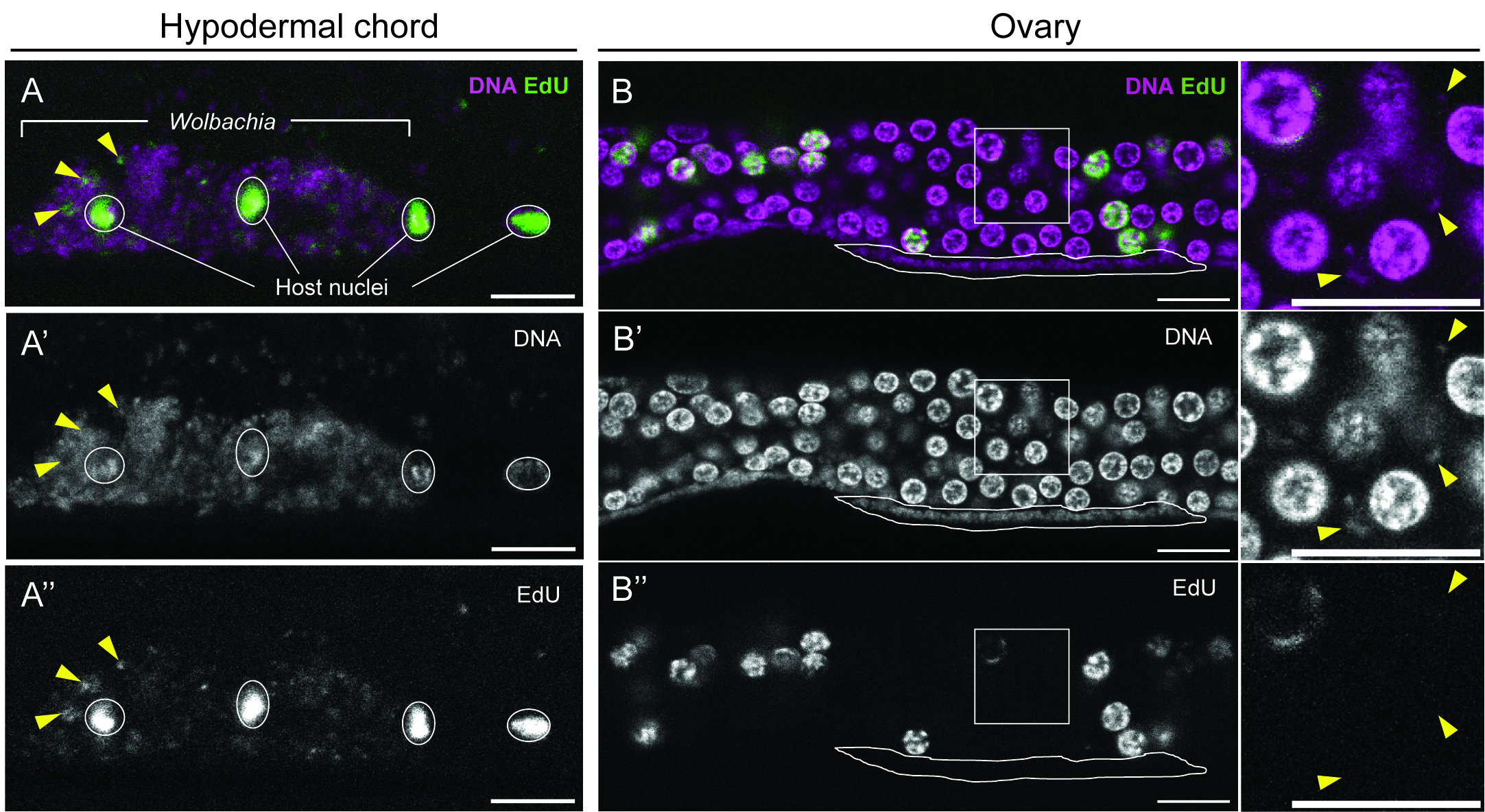

Supplement: S5 Fig — (A-A”) Hypodermal chords of adult Brugia pahangi were incubated with 200 µM EdU for 72 hours. Nematode host nuclei are outlined in white. All other magenta puncta are Wolbachia. EdU incorporation can be seen amongst the Wolbachia puncta (yellow arrowheads). Image represents a max projection of four z-stacks with a step size of 0.38 µm. DNA is stained with DAPI only. (B-B”) Ovarian tissue of adult Brugia pahangi was incubated with 200 µM EdU for 72 hours. EdU does not incorporate in Wolbachia-infected sheath cells (white dotted outline; a total of 7 infected sheath cells were analyzed). The boxed region is enlarged in the inset to the right. Nematode host oocyte nuclei incorporate EdU, but Wolbachia puncta do not (yellow arrowheads point to three representative Wolbachia puncta). DNA is stained with DAPI only. For all images, EdU is visualized with Invitrogen Click-iT EdU imaging kit, Alexa Fluor 488. All scale bars are 10 µm. (TIF) [file ppat.1012929.s006.tif]
