# Supplementary material for: Fexinidazole and Corallopyronin A target Wolbachia-infected sheath cells present in filarial nematodes
Source: PLoS Pathog. 2025 Sep 8;21(9):e1012929. doi: 10.1371/journal.ppat.1012929 (PMC12443271; doi:10.1371/journal.ppat.1012929)
Supplement: S6 Fig — The nitroreductase gene (ntr) is expressed at similar levels by wBm in three B. malayi host tissues: the proliferative zone (PZ), the meiotic zone (MZ), and the body wall (BW). (A) Raw counts of ntr and actin-like gene Wbm0154 output by featureCounts for the PZ dissection. While technical aspects can affect count number, this illustrates that the ntr gene is expressed, but at a lower level relative to actin-like Wbm0154. (B-D) Volcano plots showing similar expression of ntr across the three tissues. The Y axis is -log10 false discovery rate (FDR) and the X axis is log2 (fold change). The ntr gene is denoted in each plot. Sequencing library data were obtained from Chevignon et al. (2021). The solid black horizontal line and the vertical dashed lines denote the criteria used in Chevignon et al. (2021) for their assessment of differential gene expression: llog2(fold change)l > 2 with an FDR < 0.01. The ntr gene, like the majority in the analysis of Chevignon et al. (2021), is not differentially expressed between tissues. The relative expression of the nitroreductase is similar to expression of several other genes that include nusB (a transcription termination factor), ribosomal protein L17, and tRNA-Thr. Table indicates conserved nitroreductase genes found in the genome sequences of nine filarial nematode species. (PDF) [file ppat.1012929.s007.pdf]

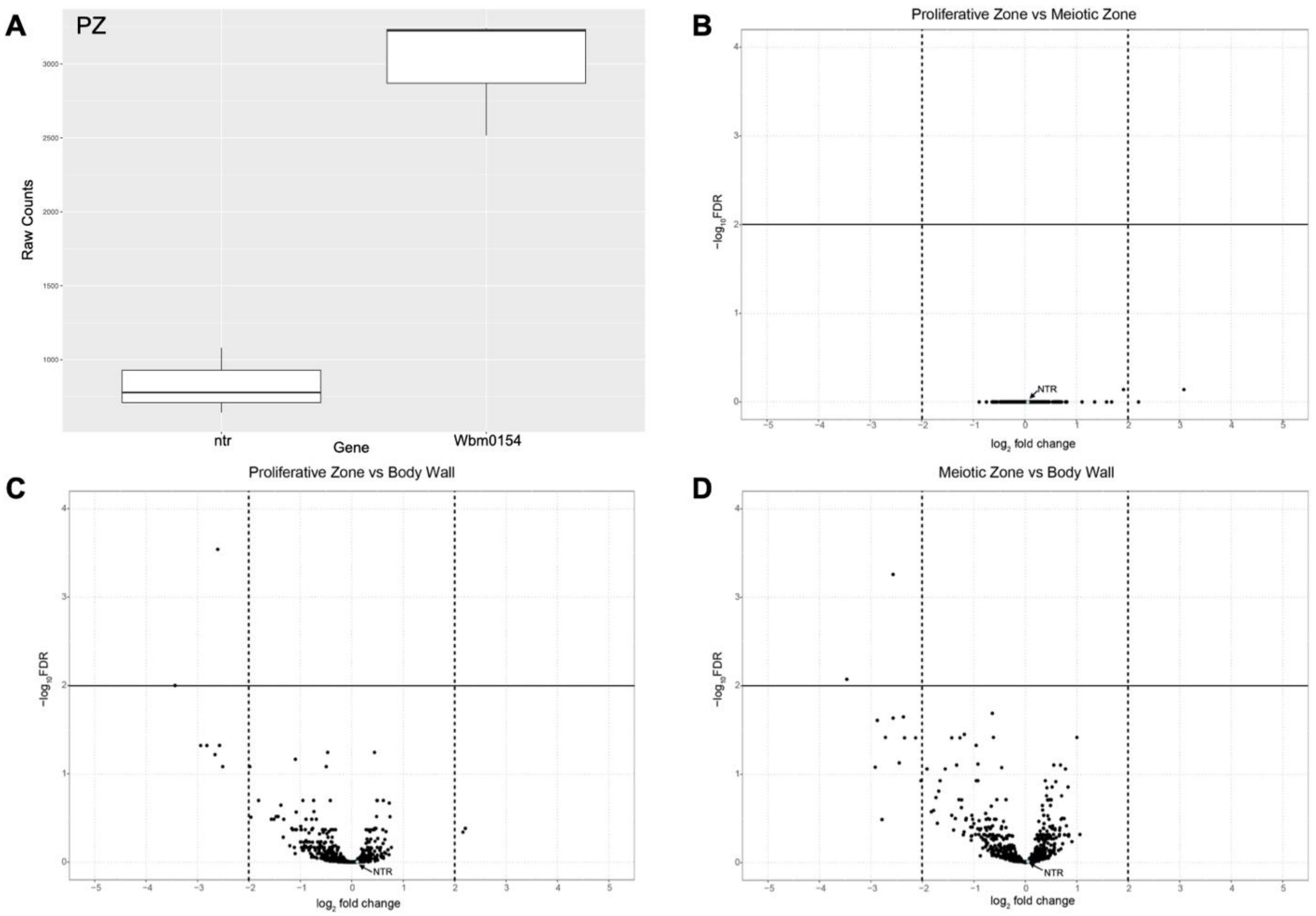

Strain	Nitrodreductase protein length	Gene accession	Protein sequence
Wolbachia endosymbiont of Brugia malayi wBm	149	WP_011256715.1	MISKEDLLELMKIRHSRRSYGQSKLAHQEEINMLIETAWLSPSRYGDEPWRQVICNRQSNQDA<br>WEKLLSCPTEPNQKWAKDTQILIISLSTKNFHDHTKIVNFWGSHDTGIANYTFMLQSYIYEFNAH<br>QMSRFDRNKIVKNSIYQMILI
Wolbachia endosymbiont of Brugia pahangi wBp	149	WP_246166039.1	MISKEDLLELMKIRHSRRSYGQSKLAHQEEINMLIETAWLSPSRYGDEPWRQVICNRQSNQDA<br>WEKLLSCPTEPNKKWAKDTQMLIISLSTKNFHDHTIKSKFLGQPRYWCSKLYIYATGYIYEFNAH<br>QMSRFDRNKIVKNSIYQMILI
Wolbachia endosymbiont of Cruorifilaria tuberocauda	187	QKX01752.1	MINTQDLLALMRIRHSGCLYDPNKVVNQEKINLLIEAARLSPSCFGDEPWRYIICNKQSNQNSW<br>VKLLNCLDESNQKWAKNAQVLIISLSAKNFRKLDKGKNFWAKHDTGAANYALILQAASANLMGH<br>QVGGFDGNKIAKKFNIPSDFNITSVIAVGYEEEGTKVNEKKRRPVEEIFFYDEWPSELI
Wolbachia endosymbiont of Dipetalonema caudispina	187	QKX01080.1	MINTQDLLALMRTRHSGCLYNPNKLVNQEEINLLIEAARLSPSCFGDEPWRYIICNKQNNQNSW<br>EKLLSCLDKSNQKWAKNAQILIISLSAKNFRELNKGKNFWAKHDTGAANYALMLQAASINLMAH<br>QVGGFNRNQIVKKFNIPNGFNITSVIAVGYEEEGAEVKEKKRRPIEEIFFYDEWPGEFI
Wolbachia endosymbiont of Dirofilaria immitis	185	WP_175818410.1	MMNTQDLLALMKIRHSGCLYDPDKVVNQEKINALIEAARLSPSCFGDEPWRYIICNKQSNQNSWKK<br>LLSCLDESNQKWAKKAQVLIISLSAKNFRKLDKGKNFWAKHDTGAANYALMLQAASTNLMAHQVG<br>GFDRNKIVKRFNIPNDF NITSVIAVGYEEEGTEVQEKKRRPIEEIFFYDEWPLS
Wolbachia endosymbiont of Litomosoides sigmodontis	185	QKX02732.1	MISKEDLLELIKIRHSGRSYDQSKLVHQKEINMLIEATRLSPSCYGDEPWRYVICNRQSNQNAWKKL<br>LSCLTEYNQKWTKDTQILIISLSAKNFRDHTKGANFWGSHDTGAANYALMLQATSMNLMAHQMGG<br>FD RDKIVKKFNIPDDFNVMSVIAIGYEKKGAEVKEKTRKPIEEIFFYNEWPKS
Wolbachia endosymbiont of Onchocerca ochengi	187	CCF78436.1	MVNTQDLLTLMKIRHSGCLYDPDKVVNQEEINSLIEAARLSPSCFGDEPWRYVICNKQSNQSSWKK<br>LLSCLNESNQKWAKNAQVLIISLSAKNFRELDKGENFWAKHDTGAANYALMLQAASTNLMAHQVG<br>GFDRNKIVKKFNIPNDFNITSVIAVGYEEEGAEIKEKKRRPVEEIFFYDEWPSELT
Wolbachia endosymbiont of Onchocerca volvulus	172	NZ_HG810405.1:4<br>74294-474856	MVNTQDLLTLMKIRHSGCLYDPDKVVNQEEINSLIEAARLSPSCFGDEPWRYVIYNKQSNQSSWKK<br>LLSCLNESNQKWAKNAQVLIISLSAKNFRELDRGENFWAKHDTGAANYALMLQAASTNLMAHQVG<br>GFDRNKIVKKFNIPNDFNITSVIAVGYEEEGAEIKEKKEGQ<br>(Frameshifted; NCBI annotated as pseudogene)
Wolbachia endosymbiont of Wuchereria bancrofti	129	OWZ25411.1	MISKEDLLELMKIRHSRHSYGQSKLAHQEEINMLIETARLSPSRYGDEPWRHVICNRQSNQDAWEK<br>LLSCPTEPNQKWAKDTQISTISLSAKNFHDHTKRANFWGSHDIGAANYTLTLQATSMNLMLIR<br>(Frameshifted; premature stop)
